# Effect of misting and wallowing cooling systems on milk yield, blood and physiological variables during heat stress in lactating Murrah buffalo

**DOI:** 10.1186/s40781-015-0082-0

**Published:** 2016-01-06

**Authors:** Brijesh Yadav, Vijay Pandey, Sarvajeet Yadav, Yajuvendra Singh, Vinod Kumar, Rajneesh Sirohi

**Affiliations:** Department of Veterinary Physiology, College of Veterinary Science and Animal Husbandry, Veterinary University, Mathura, 281001 UP India; Department of Veterinary Biochemistry, College of Veterinary Science and Animal Husbandry, Veterinary University, Mathura, 281001 UP India; Department of Livestock Production Management, College of Veterinary Science and Animal Husbandry, Veterinary University, Mathura, 281001 UP India; Department of Animal Nutrition, College of Veterinary Science and Animal Husbandry, Veterinary University, Mathura, 281001 UP India

**Keywords:** Heat stress, Buffalo, Misting, Wallowing, Milk production, Physio-biochemical responses

## Abstract

**Background:**

Heat stress adversely affects the physiological and metabolic status, and the productive performance of buffalo.

**Methods:**

The present study was conducted to explicate the effect of misting and wallowing cooling strategies during heat stress in lactating Murrah buffalo. The study was conducted for three months (May–July) of which first two months were hot dry and last month was hot humid. Eighteen lactating buffaloes, offered the same basal diet, were blocked by days in milk, milk yield and parity, and then randomly allocated to three treatments: negative control (no cooling), cooling by misting, and cooling by wallowing.

**Results:**

The results showed higher (P < 0.05) milk yield in buffaloes of misting and wallowing group compared to control during the experimental period however wallowing was found more (P < 0.05) effective during July (hot humid period). Both the treatments resulted into significant (P < 0.05) reduction in rectal temperature (RT) and respiratory rate (RR) compared to control animals during study period whereas wallowing was found to be effective on pulse rate (PR) only during July. Both treatments were resulted in mitigating the heat stress mediated decrease in packed cell volume (PCV), lymphocytopnoea and neutrophilia whereas decrease in total erythrocyte count (TEC) and monocytes was only mitigated by wallowing. Heat load induced alteration in serum creatinine and sodium concentration was significantly (P < 0.05) ameliorated by misting and wallowing whereas aspartate aminotransferase, alkaline phosphatase and superoxide dismutase activity, and reactive oxygen species concentration could be normalized neither by misting nor by wallowing. The significant (P < 0.05) increment in serum cortisol and prolactin levels observed in June and July period in control animals was significantly (P < 0.05) prevented by misting and wallowing.

**Conclusions:**

It can be concluded that misting and wallowing were equally effective in May and June (hot dry period) whereas wallowing was more effective during hot humid period in preventing a decline in milk production and maintaining physiological, metabolic, endocrine and redox homeostasis.

## Background

Buffalo (*Bubalus bubalis*) contributes 95 % of total milk production in South Asia [1] and shares 12.8 % of total milk production in spite of being only 11.6 % of the total cattle population in the world [2]. Buffalos are better converters of poor quality roughage into milk and meat and are better adapted to hot and humid climates than cattle [3]. Buffalo skin has a thick epidermis [4] which provides protection from ultra-violet rays and the secretions from well developed sebaceous glands reflect the heat rays and relieve the animal from excessive heat load [5]. However, the poor capacity for sweating due to scarcely distributed sweat glands and dark body colour, the buffaloes are less heat tolerant [6]. Buffalo are more heat stressed when they are prevented from displaying their adaptive behavioural traits such as seeking shelter, wallowing and or submerging themselves in water. In addition, high milk production results in increased production of heat in lactating buffaloes that make them most susceptible during summer as heat stress and lactation stress are combined together [7]. In Indian subcontinent (tropical and subtropical areas) exposure of animals to high ambient temperature with high humidity decrease the ability to disperse the body heat. Finally, heat stress induces increase of body temperature. The effect of heat stress has been extensively reviewed [3, 8] in buffalo and other ruminants.

The temperature humidity index (THI) is a measure of thermal comfort that has been applied to beef and dairy cattle in order to understand thermo-neutral and heat stress states. Three levels of thermal stress depending on the THI value have been explained: mild stress 72–79, moderate stress 79–89 and heavy stress >89 [9]. Cooling is recommended when THI value is 70–72 to prevent the decline in milk production [10]. When the THI value is between 72–78 a decrease in milk production may be expected in dairy cattle unless cooling strategies are applied [10]. With THI exceeding 82, cooling is indispensable [10]. Several heat stress amelioration methodologies [7, 11–14] including cooling by wallowing and sprinkling/misting [15–18] have been successfully employed to improve milk production and to expedite the process of homeostasis by evaporative heat loss mechanism. Wallowing is reported to be more effective in reducing heat stress than showering in the month of August–September [15] however little is known about the effect of cooling systems in hot dry and hot humid summer in buffalo. A wide variation is seen in ambient temperature and relative humidity during different summer months in the Indian subcontinent so effect of cooling methodologies may also differ. Therefore, the present study was designed to investigate the effects of wallowing and misting during summer months in lactating Murrah buffalo.

## Methods

### Experimental location

The experiment was carried out at the instructional livestock farm of the Veterinary University, Mathura, India which is located in the semiarid region of the country at longitude 78°E and latitude 27°N and at an altitude of 176 m above mean sea level. The average annual maximum and minimum ambient temperature ranges from 5 to 45 °C. The mean annual relative humidity ranges from 25 to 85 %. The annual rainfall in this area ranges from 200 to 400 mm with an erratic distribution throughout the year. The experiment was carried out during the month of May to July when average temperature and THI ranged between 32–36 °C and 73–90, respectively. Daily minimum and maximum temperature and relative humidity was recorded during the experimental period and THI was calculated by the formula [19].

$$ \mathrm{T}\mathrm{H}\mathrm{I} = \left(0.8 \times {\mathrm{T}}_{\mathrm{db}}\right) + \left[\left(\mathrm{R}\mathrm{H}/100\right) \times \left({\mathrm{T}}_{\mathrm{db}} - 14.4\right)\right] + 46.4 $$

T_db_- Dry bulb temperature, RH- Relative humidity

### Experimental animals and management

Eighteen healthy lactating Murrah buffalo based on their lactation number, stage of lactation, body weight, dam’s highest milk yield and milk yield in current lactation were selected for this study. The animals were provided balanced ration as per the recommendations [20] and clean drinking water *ad libitum* and the ration was kept constant during period of the experiment. The detailed ingredients and composition of ration is presented in Table 1. Prophylactic measures against foot and mouth disease, hemorrhagic septicemia, endo and ecto-parasitic infestations were carried out as prescribed by the health calendar of the farm to ensure that the animals were in a healthy condition throughout the study. The animals were housed in well ventilated sheds made of asbestos roofing and cement concrete floor with the slopes towards the drain to avoid dampness. The animals of the treatment groups were kept in different sheds. The animals were housed in tail to tail housing system where the rear wall of the manger was about 1 m and above the wall about 1.5 m was open for proper ventilation. The shed is fitted with a misting systems and ceiling fans. Misting was applied in an area of 72 m^2^ with the help of eight misting pumps, each having a capacity to use 1.5 l of water per minute with a droplet size of 3–5 micron. A water tank of 15x10x2.5 m dimension, filled up to 2.25 m with water volume of 337500 l was provided for wallowing and the tank was washed, emptied and refilled at three days interval. The spent water was used for irrigating the land used for fodder cultivation. The research protocol for this study was approved by institutional animal ethics committee, Veterinary University Mathura, India.

**Table 1:** Ingredient and nutrient composition of diets fed to lactating buffaloes

Attribute	Percent
Ingredients (%)	
Wheat straw	20.0
Green fodder	38.0
Barley grain	16.0
Wheat grain	4.0
Wheat bran	8.0
Mustard oil cake	14.2
Mineral mixture	0.80^*^
Composition (%)	
Dry Matter	63.42
Organic Matter	90.54
Ether Extract	2.78
Crude Protein	12.62
Total Ash	9.46
Nitrogen Free Extract	56.14
NDF	46.11
ADF	27.67
Hemicellulose	20.45
ADL	11.28
Cellulose	14.38
Ca	0.72
P	0.48

^*^Contained CaHPO4 (40 %); CaCO_3_ (30 %); NaCl (22 %); FeSO_4_ (2.5 %); CuSO_4_ (2.1 %); ZnSO_4_ (0.75 %); CoCl_2_ (0.05 %); MnCl_2_ (0.6 %); MgSO_4_ (2.0 %); KI (0.01 %)

### Experimental design

**Fig. 1:**
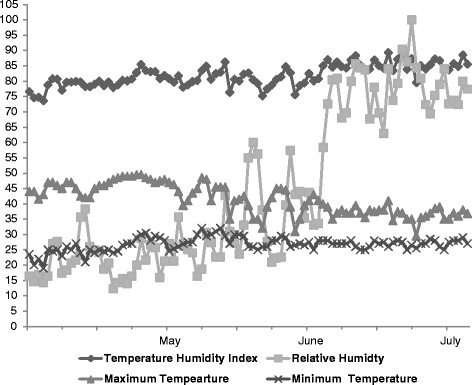
Daily minimum and maximum temperature (°C), relative humidity (%) and temperature humidity index during the experimental period (May–July)

The study was conducted during May to July 2013 when ambient THI remained high whereas the relative humidity was low and mean environmental temperature was high during May and June, and on contrary relative humidity was high and mean environmental temperature was comparatively lower in July (Fig. 1). Eighteen lactating buffalos were divided into three equal groups of six animals and given three different treatments: negative control (without any cooling), T1 (misting) (misting was done for 10 min at the interval of every half an hour from 11:30 h to 15:30 h) and T2 (wallowing) (the animals were allowed for wallowing twice a day for an hour each i.e. from 11:30 to 12:30 h and 14:30 to 15:30 h). Milking was done manually and milk yield was recorded twice a day at 06:00 and 18:00 h. Physiological parameters were recorded and blood was sampled (n = 18) just after treatment on the 30^th^ day of each month at 14:30 h. The blood samples were collected in duplicate; one with anticoagulant for hematological examinations and another without anticoagulant for harvesting the serum for biochemical and hormonal estimations.

### Physiological Parameters

Rectal temperatures (RT) were measured using a clinical thermometer and expressed in °C. Respiratory rates (RR) were recorded by observing costal movements during 30 s [21] and presented as breathes per minute. The pulse rate (PR) of the animals was recorded by observing the pulsation of the middle coccygeal artery at the base of the tail and expressed as beats per minute.

### Blood Collection and Serum Separation

Eight millilitres blood was collected from the external jugular vein using 18 gauge sterilized disposable needles and plastic syringes in duplicate one with ethylene diamine tetraacetic acid (EDTA) anticoagulant and another without anticoagulant. The blood samples with anticoagulant were used for hematological examination, while others without anticoagulant were used for serum separation. Serum was harvested from blood after complete clotting followed by centrifugation at 3,500 rpm at room temperature for 20 min and after separation kept at −20 °C till further analysis.

### Analysis of Feed

Feed samples were also analyzed for the proximate principles [22], neutral detergent fiber (NDF) and acid detergent fiber (ADF) [23], calcium [24] and phosphorous [25].

### Hematological parameters

The collected whole blood samples were analyzed for hematological parameters such as total erythrocyte count (TEC), packed cell volume (PCV), hemoglobin (Hb), total leukocyte count (TLC) by standard methods. The erythrocyte indices (mean corpuscular volume (MCV), mean corpuscular hemoglobin (MCH) and mean corpuscular hemoglobin concentration (MCHC)) was calculated by the standard formulae [26]. Blood films were prepared and stained with Lieshman solution, and used for differential leukocyte count (percent neutrophil, lymphocyte, eosinophil, monocyte and basophil).

### Biochemical Parameters

Serum aspartate aminotransferase and alkaline phosphatase activity, urea, creatinine, glucose, calcium, phosphorous and chloride were analyzed using commercially available kits (Cogent, clinical chemistry division of SPAN diagnostic Ltd, India). The sodium and potassium concentration in the serum was measured by flame photometry. Superoxide dismutase (SOD) activity in serum was measured using microtitre plate method [27]. One unit of SOD was defined as the amount of protein required to inhibit the MTT reduction by 50 %. The reactive oxygen species (ROS) level was estimated in terms of hydroxyl radical (HR) in serum samples [28].

### Hormone Assay

Serum cortisol, tri-iodothyronine (T_3_), thyroxin (T_4_) and prolactin concentrations were estimated by ELISA kits supplied by Fisher Scientific (Thermo Fisher Scientific India, Private Limited). The intra assay variability for cortisol, prolactin, T_3_ and T_4_ were less than 8, 4.03, 10.7 and 8.16 % respectively whereas the inter assay variability were less than 15, 5.49, 9.1 and 8.42 % respectively.

### Statistical Analysis

The effect of summer months on environmental variables was analysed using ANOVA model (SAS, 9.4). The effects of month, treatment and interaction of month and treatment on milk production, physiological, biochemical and endocrine parameters were analyzed using repeated measures of ANOVA model (SAS, 9.4). Differences among the months, treatments and interaction of month and treatment were determined using Tukey's test (SAS, 9.4) and indicated by both *p values* and superscripts (*P* < 0.05). Least squares means and pooled standard errors were reported. The level of significance was set at *P* < 0.05.

## Results

### Meteorological Conditions

The average maximum and minimum temperature (°C), average temperature, relative humidity and THI during the experimental period if presented in Table 2.

**Table 2:** Average environmental temperature, relative humidity (RH) and temperature humidity index (THI) during the experimental period

Environmental Variables	May	June	July	SE	*P* Value
Minimum Temperature	25.60 ^a^	28.02 ^b^	26.91 ^b^	0.51	<.0001*
Maximum Temperature	46.46 ^a^	41.08 ^b^	36.83 ^c^	0.82	<.0001*
Average Temperature	36.03 ^a^	34.55 ^b^	31.88 ^c^	0.61	<.0001*
Relative Humidity	21.67 ^a^	33.53 ^b^	77.32 ^c^	2.41	<.0001*
THI	79.88^a^	80.57 ^a^	85.36 ^b^	0.60	<.0001*

Means within a row having different superscripts differ significantly (*p* < 0.05)

The standard error uses pooled estimate of error variance

### Milk Production

**Fig. 2:**
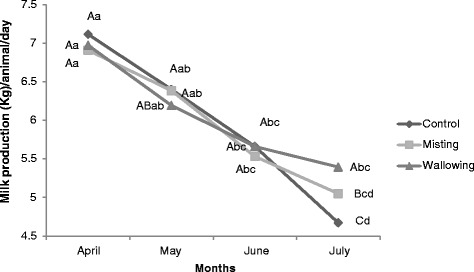
Effect of misting and wallowing on milk production in heat stressed lactating Murrah buffaloes during the experimental period. Values with different upper case letters at the same experimental month and values with different lower case letter between the experimental months differ significantly (*P* < 0.05)

For this experiment, every group of the animals had similar milk production at the beginning of the experiment. There was a steep decline in milk production in control group during the experimental period whereas in misting group milk production did not decrease significantly (*P* > 0.05) during May and June (hot dry period) but milk production decreased significantly (*P* < 0.05) in July (hot humid period) as compared to May (Fig. 2). In wallowing group there was no significant (*P* > 0.05) decline in milk production during the experimental period.

### Physiological Parameters

The effect of misting and wallowing on physiological parameters in lactating Murrah buffalo is presented in Table 3. The significant (*P* < 0.05) differences were observed in different physiological parameters during the experimental period. The RT and RR were significantly (*P* < 0.05) lower in misting and wallowing group as compared to control during the experimental period whereas PR was significantly (*P* < 0.05) lower only in wallowing group as compared to control only in July. In May and June the misting and wallowing showed similar significant (*P* < 0.05) effects on RT and RR however wallowing was significantly (*P* < 0.05) effective in preventing an increase in RT in July.

**Table 3:** Effect of misting and wallowing on physiologic parameters of heat stressed lactating Murrah buffaloes

Parameters		RT	RR	PR
Period (*p* value)		<.0001*	<.0001*	<.0001*
Treatment (*p* value)		<.0001*	<.0001*	<.0001*
Control		38.81^a^	36.61^a^	48.56 ^a^
Misting		38.41 ^c^	31.67 ^b^	49.06 ^b^
Wallowing		38.57 ^b^	32.39 ^b^	50.61 ^b^
SE for Treatment		0.03	0.24	0.23
Period*Treatment (*p* value)		<.0001*	0.07	0.002*
May	Control	38.65 ^bc^	34.00 ^b^	50.00 ^ab^
	Misting	38.28 ^d^	30.17 ^e^	50.00 ^ab^
	Wallowing	38.30 ^d^	31.00 ^de^	51.00 ^a^
June	Control	38.78 ^b^	38.83 ^a^	49.00 ^ab^
	Misting	38.48 ^cd^	33.33 ^bc^	49.00 ^ab^
	Wallowing	38.45 ^cd^	33.67 ^b^	49.83 ^ab^
July	Control	39.30 ^a^	37.00 ^a^	46.67 ^c^
	Misting	38.73 ^b^	32.50 ^bcd^	48.17 ^bc^
	Wallowing	38.40 ^d^	31.50 ^cde^	51.00 ^a^
SE for Period*Treatment		0.05	0.03	0.41

Effect of Period, treatment and interaction of Period and treatment are indicated with *p* values

Means within a column having different superscripts differ significantly (*p* < 0.05)

The standard error uses pooled estimate of error variance

*RT* Rectal Temperature (°C), *RR* Respiratory rate (breathes/minute), *PR* Pulse Rate (beats/minute)

### Hematological parameters

A significant (*P* < 0.05) change was observed in neutrophil and lymphocyte percentage during different periods of experiment (Table 4). The PCV and lymphocyte count was significantly (*P* < 0.05) higher and neutrophil percentage was significantly (*P* < 0.05) lower in both misting and wallowing groups as compared to control whereas TEC and monocyte percentage was significantly (*P* < 0.05) higher only in wallowing group as compared to control (Table 4). The TLC was significantly (*P* < 0.05) lower in misting and wallowing group as compared to control during the experimental period. The neutrophil percentage was significantly (*P* < 0.05) reduced in misting and wallowing group as compared to control during different periods of experiment however in control group the neutrophil percentage was significantly (*P* < 0.05) higher in July as compared to May and June and a similar but opposite trend was observed with respect to lymphocyte percentage. Monocyte percentage was significantly (*P* < 0.05) higher in wallowing group as compared to control however it was not affected by misting.

**Table 4:** Effect of misting and wallowing on hematological parameters of heat stressed lactating Murrah buffaloes

Parameters		PCV (%)	Hb (g %)	TEC (10^6^/μl)	MCV (fL)	MCH (pg)	MCHC	TLC (10^3^/μl)	Neutro %	Lymph %	Mono %	Eosino %	Baso %
Period (*p* value)		0.977	0.77	0.51	0.47	0.64	0.77	0.20	0.01*	0.002*	0.82	0.77	0
Treatment (*p* value)		0.01*	0.12	0.03*	0.06	0.08	0.06	<.0001*	<.0001*	<.0001*	<.0001*	0.63	0
Control		25.42^b^	9.17	4.63 ^b^	55.40	19.77	36.53	7.40^a^	32.94 ^a^	57.94 ^a^	4.11^a^	5.00	0
Misting		29.79 ^a^	9.72	4.91 ^ab^	51.47	19.76	32.81	7.03^b^	27.83 ^b^	62.83 ^b^	4.17^a^	5.17	0
Wallowing		30.45 ^a^	10.33	5.30 ^a^	60.64	17.48	33.94	6.99^b^	26.61 ^b^	63.50 ^b^	5.17^b^	4.72	0
SE for Treatment		1.17	0.39	0.17	2.61	0.81	1.11	0.03	0.43	0.38	0.14	0.33	0
Period*Treatment (*p* value)		0.96	0.99	0.92	0.60	0.64	0.86	0.10	0.01*	0.0004*	0.93	0.87	0
May	Control	26.33	9.40	4.82	57.88	19.46	36.32	7.25 ^ab^	31.00 ^b^	60.00 ^cd^	4.00 ^b^	5.00	0
	Misting	29.22	10.07	5.11	64.34	19.62	34.84	6.99^bc^	26.67^cd^	64.17^a^	4.17 ^ab^	5.00	0
	Wallowing	30.65	10.43	5.36	60.52	16.47	34.06	7.03 ^bc^	26.33 ^cd^	63.50 ^ab^	5.17 ^a^	5.00	0
June	Control	25.61	9.23	4.63	55.33	19.87	36.45	7.44 ^a^	32.67 ^ab^	58.17 ^de^	4.00 ^b^	5.17	0
	Misting	30.00	9.60	4.94	57.05	19.38	32.07	7.02 ^bc^	29.50 ^bc^	60.67 ^bcd^	4.17 ^ab^	5.67	0
	Wallowing	30.05	10.13	5.18	55.75	19.51	33.70	7.08 ^bc^	27.50 ^cd^	62.83 ^abc^	5.17 ^a^	4.50	0
July	Control	24.33	8.87	4.43	48.27	19.97	36.85	7.52^a^	35.17 ^a^	55.67 ^e^	4.33 ^ab^	4.83	0
	Misting	30.16	9.50	4.68	55.13	20.29	31.51	6.95 ^c^	27.33 ^cd^	63.67 ^ab^	4.17 ^ab^	4.83	0
	Wallowing	30.65	10.43	5.36	48.27	16.47	34.06	7.01 ^bc^	26.00 ^d^	64.17^a^	5.17 ^a^	4.67	0
SE for Period*Treatment		2.02	0.67	0.29	4.53	1.40	1.92	0.60	0.74	0.66	0.26	0.57	0

Effect of Period, treatment and interaction of Period and treatment are indicated with *p* values

Means within a column having different superscripts differ significantly

The standard error uses pooled estimate of error variance

*PCV* Packed Cell Volume, *Hb* Hemoglobin, *TEC* Total Erythrocyte Count, *MCV* Mean Corpuscular Volume, *MCH* Mean Corpuscular Hemoglobin, *MCHC* Mean Corpuscular Hemoglobin Concentration, *TLC* Total Leukocyte Count, *Neutro* Neutrophil, *Mono* Monocyte, *Eosino* Eosinophil, *Baso* Basophil

### Metabolic parameters

The serum urea, creatinine, potassium, AST, AKP, SOD and ROS concentration changed significantly (*P* < 0.05) during different experimental periods (Table 5). The creatinine concentration in serum was significantly (*P* < 0.05) lower in wallowing group whereas sodium concentration was significantly (*P* < 0.05) higher in both misting and wallowing group as compared to control. Serum urea concentration increased significantly (*P* < 0.05) in July as compared to May in control group however misting and wallowing did not show significant (*P* > 0.05) effect on urea concentration in different periods of experiment (Table 5). The sodium concentration was significantly (*P* < 0.05) higher in both misting and wallowing group as compared to control during all the periods of the experiment whereas only wallowing was significantly (*P* < 0.05) effective in maintaining normal potassium levels. A significant increase in serum AKP activity and ROS concentration was observed in all the groups during July indicating that wallowing and misting was not able to minimize the increase in AKP activity and ROS concentration. A significant (*P* < 0.05) increase in AST activity was also observed in misting group in July as compared to May.

**Table 5:** Effect of misting and wallowing on serum metabolites, electrolytes, enzyme activity and redox status of heat stressed lactating Murrah buffaloes

Parameters		Urea	Creatinine	Glucose	Sodium	Potassium	Chloride	AST	AKP	SOD	ROS
Period (*p* value)		<.0001*	<.0001*	0.1132	0.15	0.0203*	0.26	<.0001*	<.0001*	0.0054*	<.0001*
Treatment (*p* value)		0.33	0.02*	0.2298	<.0001*	0.52	0.99	0.4618	0.95	0.48	0.65
Control		20.27	1.87^a^	47.49	129.94 ^b^	6.72	89.51	40.20	10.01	304.6	4.86
Misting		20.19	1.80 ^b^	48.07	153.83 ^a^	6.56	89.45	39.40	10.05	314.49	4.80
Wallowing		19.94	1.84 ^ab^	47.78	152.83 ^a^	6.89	89.47	39.88	10.03	315.16	4.86
SE for Treatment		0.16	0.02	0.23	0.70	0.20	0.27	0.45	0.10	6.79	0.05
Period*Treatment (*p* value)		0.94	0.64	0.30	0.04*	0.003*	0.99	0.71	0.68	0.35	0.64
May	Control	19.53 ^d^	1.91 ^d^	47.53	129.17 ^b^	5.33 ^b^	89.13	39.99 ^ab^	10.23 ^a^	308.80	4.56 ^b^
	Misting	19.40^d^	1.78 ^d^	47.98	154.83 ^a^	6.50 ^ab^	89.10	38.03^b^	10.38^a^	305.60	4.60^b^
	Wallowing	19.70^cd^	1.84 ^cd^	47.93	151.5 ^a^	7.17 ^a^	89.10	38.72 ^ab^	10.43 ^a^	305.80	4.71 ^b^
June	Control	20.03 ^abcd^	1.77 ^abcd^	48.33	128.67 ^b^	7.17 ^a^	89.60	39.10 ^ab^	10.62 ^a^	298.90	4.69 ^b^
	Misting	19.53 ^d^	1.70 ^d^	48.20	151.17 ^a^	6.33 ^ab^	89.53	38.53 ^ab^	10.42 ^a^	297.70	4.57 ^b^
	Wallowing	19.90 ^bcd^	1.75 ^bcd^	47.80	154.50 ^a^	6.50 ^ab^	89.67	38.93 ^ab^	10.54 ^a^	296.83	4.63 ^b^
July	Control	21.00 ^ab^	1.93 ^a^	46.60	132.0 ^b^	7.67^a^	89.80	41.50^ab^	9.18 ^b^	306.1	5.34 ^a^
	Misting	20.90 ^abc^	1.90 ^abc^	48.02	155.50 ^a^	6.83 ^ab^	89.72	41.65 ^a^	9.36^b^	340.17	5.25^a^
	Wallowing	21.20 ^a^	1.93 ^a^	47.60	152.50 ^a^	7.00 ^a^	89.63	42.00 ^a^	9.11 ^b^	342.83	5.25 ^a^
SE for Period* Treatment		0.27	0.03	0.41	1.22	0.35	0.47	0.78	0.18	11.76	.08

Effect of Period, treatment and interaction of Period and treatment are indicated with *p* values

Means within a column having different superscripts differ significantly

Urea (mg/100 ml), Creatinine (mg/100 ml), Glucose (mg/100 ml), Sodium (meq/L), Potassium (meq/L), Chloride (mg/100 ml), *AST* Aspartate aminotransferase (IU/L), *AKP*Alkaline phosphatase (KA units), *SOD* Superoxide dismutase (U/ml), *ROS* Reactive oxygen species (mg H_2_O_2_ equivalents/ml)

### Endocrine parameters

The effect of misting and wallowing on serum cortisol, prolactin, T_3_ and T_4_ during summer stress in lactating buffalo is presented in Table 6. The serum cortisol level increased significantly (*P* < 0.05) in control group during July period as compared to May and June however, cortisol level decreased significantly (*P* < 0.05) in misting and wallowing groups during high THI period. The serum prolactin concentration increased significantly (*P* < 0.05) in July period in all the groups as compared to May and June. Serum prolactin level decreased significantly (*P* < 0.05) in wallowing group in May whereas, both misting and wallowing were able to significantly (*P* < 0.05) decrease the prolactin level in July. The T_3_ and T_4_ levels did not change significantly (*P* > 0.05) in all the groups throughout the experimental period.

**Table 6:** Effect of misting and wallowing on endocrine parameters of heat stressed lactating Murrah buffaloes

Parameters		Cortisol	Prolactin	T_3_	T_4_
		(ng/ml)	(ng/ml)	(ng/ml)	(ng/ml)
Period (*p* value)		<.0001*	<.0001*	0.0537	0.0003*
Treatment (*p* value)		0.0009*	<.0001*	0.0527	0.9234
Control		3.34 ^c^	83.83^c^	1.67	38.82
Misting		2.83^a^	57.52^a^	1.35	39.16
Wallowing		3.15 ^b^	76.54^b^	1.73	38.04
SE for Treatment		0.14	5.59	0.07	1.31
Period*Treatment (*p* value)		<.0001*	<.0001*	0.3300	0.9091
May	Control	2.27 ^cd^	55.25 ^cd^	1.61	45.98
	Misting	2.23^cd^	39.71^de^	1.59	46.74
	Wallowing	2.19 ^d^	34.19 ^e^	2.05	44.35
June	Control	2.79 ^bcd^	45.82 ^cde^	1.51	31.54
	Misting	3.31 ^b^	55.56 ^cd^	1.24	35.17
	Wallowing	2.81 ^bcd^	59.29 ^bc^	1.32	33.17
July	Control	4.96 ^a^	150.4^a^	1.88	35.30
	Misting	2.94 ^bc^	77.29 ^b^	1.23	37.57
	Wallowing	4.44 ^a^	136.13 ^b^	1.82	38.23
SE for Period*Treatment		0.16	4.06	0.19	3.52

Effect of Period, treatment and interaction of Period and treatment are indicated with *p* values

Means within a column having different superscripts differ significantly (*p* < 0.05)

The standard error uses pooled estimate of error variance

## Discussion

The magnitude of heat stress is defined by sum of different abiotic environmental forces which is combined effect of dry bulb temperature (dbT), relative humidity (RH), solar radiation and wind speed [3]. In the present experiment the relative humidity was very low and environmental temperature was high during May and June which facilitated better evaporative cooling whereas in July, comparatively less dry bulb temperature but very high relative humidity reduced the rate of evaporative cooling.

A thermal environment is a major factor that can negatively affect milk production in buffaloes however the parity, stage of lactation [29], breeds and milk yield of the animal [30] also influence milk production. In the present experiment the parity, breed and milk yield of the animals was similar for each treatment group. The present study showed a significant gradual decrease in milk production during the experimental period. In spite of advancing lactation it was observed that wallowing and misting were equally effective in preventing a decline in milk production during May and June (hot dry period) however, wallowing was highly effective during July (Hot humid period) in maintaining milk production. Similar amelioration effects on milk production by misting was also reported [31] in buffaloes and Holstein Friesian cows [32], substantiates the findings of present study. Wallowing was more effective because besides evaporative heat loss, conductive and convective heat loss also prevailed during wallowing that helped in additional heat loss from buffalo.

Performance of animals is a great challenge in tropical and subtropical region due to high ambient temperature especially in summer months where ambient temperature increases by more than 4 °C as compared to the normal environmental temperature [33]. In present study, the animals of control group responded to increased heat load due high temperature humidity index (THI) by increasing physiological heat loss mechanism, *viz.* respiratory rate and pulse rate as in previous reports [7, 12, 17, 18, 34, 35]. A significant correlation between THI and physiological parameters such as RT, RR and PR was also reported [34] supporting the findings of present study. The response changes in physiological parameters are results of adaptive mechanisms of animal in an attempt to restore its thermal balance. The increase in respiration rate was observed to be related with discomfort and mainly due to exposure to greater intensity heat stress [6]. At higher temperature, the respiration rate increased rapidly to about 3 to 4 times the normal values in buffaloes [37]. However these mechanisms were not sufficient enough to reduce the heat load of the body that resulted in increase in rectal temperature of the animals as reported in previous studies [35, 38, 39] . In contrast, misting and wallowing were able to prevent the increase in RR, PR and RT by decreasing the heat load of the body by enforcing additional physical heat loss from the body which is also supported by previous studies [7, 15, 17] in different breeds of buffaloes. We also observed that wallowing was more effective as compared to misting in decreasing the RT because misting involves heat loss only by evaporative cooling whereas wallowing involves heat loss by both evaporative cooling and conduction as the low hair density on skin helps in readily exchange of heat from skin to water. The thickness of buffalo skin hinders heat dissipation through convection and radiation but this limitation is overcome by wallowing. Similar reports were also observed [15] which corroborated the present findings. Our results also indicated that misting and wallowing both were equally effective as a cooling strategy during hot dry period however, during very hot humid period wallowing was more effective in decreasing the heat load from the animal body. Wallowing was reported to be more effective in preventing an increase in physiological parameters in hot humid summer stress [15]. In present study, during hot dry period the temperature gradient between the animal body and environment was supportive for evaporative cooling however, in hot humid period the temperature gradient was similar to the hot dry period but high RH of the micro-environment restricted the heat loss by evaporatory cooling and therefore wallowing proved to be more effective strategy as heat loss mechanism as it involved heat loss by conduction and even convection besides evaporative cooling.

The present experiment showed that misting and wallowing were equally effective in preventing a change in various hematological parameters (TEC, PCV, TLC, Neutrophil % and lymphocyte %) during different THI periods in buffalo. The TEC and PCV was significantly lower in control group as compared to misting and wallowing group because heat stress led to increased water intake [39] which caused hemo-dilution [40], resulting in decreased TEC and PCV in control group however animals of misting and wallowing group cooled themselves effectively and prevented subsequent alterations in hematological parameters [18]. Neutrophilia and lecocytopnoea i.e. increased neutrophil/ lymphocyte ratio [41, 42] was also observed in control animals which attributed to increased glucocorticoids [18, 43] mediated alterations in the redistribution of lymphocytes from the blood to other body compartments [44] during heat stress. At the same time, glucocorticoids also increase an influx of neutrophils into the blood circulation from bone marrow and also reduce the migration of neutrophils from the blood to other compartments [45]. However, misting and wallowing successfully ameliorated the heat stress and resulted in no change in cortisol mediated alterations in neutrophil and lymphocyte ratio.

Serum urea level is very variable, depending on gluconeogenesis (protein degradation), catabolism of amino acids and rumen ammonia levels [3]. In present study serum urea and creatinine level increased during hot humid period in all the experimental animals [36] as the animals experienced more intense heat load in hot humid period. The heat load contributed to protein degradation using it as substrate for gluconeogenesis for energy production to maintain euthermia that resulted in an increased serum urea and creatinine level in control group however the increase in misting and wallowing group could not be justified. The concentration of major serum electrolytes (sodium, potassium and chloride) were affected during summer months in buffalo [11, 13]. In present experiment the level of sodium was lower in control animals during the experiment as compared to treatment groups whereas potassium level was lower only in hot dry period and chloride level did not change. Similar effect of heat stress was reported [13, 18] in Murrah buffalo heifers. The alteration in the electrolyte concentration is attributed to the loss of electrolytes during sweating to cool the body during heat stress however, in both treatment group electrolyte level did not change because sweating did not occur to loss the heat from body as misting and wallowing were able to minimize the heat load of the animal. Sprinkling was reported to be helpful in maintaining normal sodium and potassium levels [18]. However, it was suggested that wallowing was more effective than spraying in maintaining electrolyte balance during summer stress in buffalo [46].

Serum aspartate aminotransferase (AST) and alkaline phosphatase (AKP) activity has been used to investigate the effect of different heat stress ameliorative measures in buffalo [13, 18]. Heat stress caused an increase in AST and decrease in AKP activity [13, 18] however, in present experiment, the same trend was observed only during hot humid period as compared to hot dry period for all the group of animals which indicated that animals were affected more by increased heat load during hot humid period. Further results indicated that misting and wallowing were not effective in maintaining normal serum AST and AKP activity. The similar non significant effects of sprinkling on AST activity has been also reported [18].

Redox status of the animal cell get disturbed under hyperthermic conditions both in-vivo [11, 47, 48] and in-vitro [49] and culminates in oxidative stress [50]. In present experiment superoxide dismutase (SOD) activity and reactive oxygen species (ROS) level in serum was estimated to quantify the systemic antioxidative defense when animals were cooled by misting and wallowing. It was observed that SOD activity and ROS level tended to increase in hot humid period as compared to hot dry period in all the groups and consequently escalated production of oxidative free radical was observed. A similar increase in SOD activity after exposure to heat stress also reported in buffaloes, simulates the findings of present study [11, 47]. To counter balance the redox potential, SOD activity improved marginally. It was also observed that intrinsic antioxidative defense system of the animals was sufficient enough to neutralize the increased ROS levels however; misting and wallowing could not mitigate the ROS production.

Heat stress acclimation is accomplished by an altered endocrine status that ultimately affects target tissue responsiveness to environmental stimuli. Hormones implicated in the acclamatory response to heat stress primarily include thyroid hormones [39, 43], prolactin [51] and glucocorticoids [11, 39, 43]. In present study, the peak cortisol and prolactin level in control animals during hot humid period as compared to hot dry period indicated that hot humid period was more stressful to the lactating buffalo. Increase in cortisol [39, 43] and prolactin [51] has been earlier established in buffalo during heat stress. In present study, a decreasing trend in T4 level was observed in hot humid period in order to decrease basal metabolic rate to decrease metabolic heat production. A decrease in T3 and T4 levels has been also reported in buffalo in response to summer heat stress [35, 43, 52]. Misting and wallowing was observed equally effective in preventing an increase in cortisol and prolactin level in high THI period however, it was reported that wallowing was more effective cooling strategy than sprinkling [16] whereas no change in cortisol and thyroid hormone levels was observed after application of cooling and other heat stress amelioration strategies in Murrah buffaoes [7].

## Conclusions

The temperature humidity index based on the recorded temperature and humidity during this trial indicated that micro-environment for the buffaloes were stressful as highlighted by alteration in milk production performance and animal responses (rectal temperature, respiratory rate, pulse rate, hematological parameters, serum metabolites, electrolytes, enzyme activities, redox status and stress hormones) in control group. Misting and wallowing proved to be equally effective as a cooling strategy during May and June (Hot-dry) period by preventing an alteration in physio-biochemical and endocrine response, and a decline in milk production whereas wallowing was more effective during July (hot-humid) period.

## References

[1] Javaid SB, Gadahi JA, Khaskeli M, Bhutto MB, Kumbher S, Panhwar AH (2009). Physical and chemical quality of market milk sold at Tandojam, Pakistan. Pak Vet J.

[2] FAOSTAT. http://faostat.fao.org/. 2007. Accessed on 12th September 2015.

[3] Marai IFM, Haeeb AAM (2010). Buffalo's biological functions as affected by heat stress—A review. Livest Sci.

[4] Shafie MM, Yousef MK (1985). Physiology responses and adaptation of water buffalo. Stress Physiology in Livestock Vol II, Ungulates’.

[5] Shafie MM, El-Khair MA (1970). Activity of the sebaceous glands of bovines in hot climates. United Arab Republic J Anim Prod.

[6] Das SK, Upadhyay RC, Madan ML (1999). Heat stress in Murrah buffalo calves. Livest Prod Sci.

[7] Das KS, Singh J, Singh G, Upadhyay R, Malik R, Oberoi P (2014). Heat stress alleviation in lactating buffaloes: Effect on physiological response, metabolic hormone, milk production and composition. Indian J Anim Sci.

[8] Yadav B, Singh G, Verma AK, Dutta N, Sejian V (2013). Impact of heat stress on rumen functions. Vet World.

[9] Akyuz A, Boyaci S, Cayli A (2010). Determination of critical period for dairy cows using temperature humidity index. J Anim Vet Adv.

[10] Broucek J, Novak P, Vokralova J, Soch M, Kisac P, Uhrinca M (2009). Effect of high temperature on milk production of cows from free-stall housing with natural ventilation. Slovak J Anim Sci.

[11] Kumar BVS, Singh G, Meur SK (2010). Effects of addition of electrolyte and ascorbic acid in feed during heat stress in buffaloes. Asian Australas J Anim Sci.

[12] Singh SP, Hooda OK, Kumar P (2011). Effect of yeast supplementation on feed intake and thermal stress mitigation in buffaloes. Indian J Anim Sci.

[13] Singh SP, Hooda OK, Kundu SS, Singh S (2012). Biochemical changes in heat exposed buffalo heifers supplemented with yeast. Trop Anim Health Prod.

[14] Kumar M, Kaur H, Tyagi A, Mani V, Deka RS, Chandra G (2013). Assessment of chromium content of feedstuffs, their estimated requirement, and effects of dietary chromium supplementation on nutrient utilization, growth performance, and mineral balance in summer-exposed buffalo calves (Bubalus bubalis). Biol Trace Elem Res.

[15] Aggarwal A, Singh M (2008). Changes in skin and rectal temperature in lactating buffaloes provided with showers and wallowing during hot-dry season. Trop Anim Health Prod.

[16] Aggarwal A, Singh M (2010). Hormonal changes in heat-stressed Murrah buffaloes under two different cooling systems. Buffalo Bull.

[17] Rahangdale PB, Ambulkar DR, Somnathe RD (2011). Influence of summer managemental practices on physiological responses and temperament in murrah buffaloes. Buffalo Bull.

[18] Vijayakumar P, Dutt T, Singh M, Pandey HN (2011). Effect of heat ameliorative measures on the biochemical and hormonal responses of buffalo heifers. J Appl Anim Res.

[19] Mader TL, Davis MS, Brown-Brandl T (2006). Environmental factors influencing heat stress in feedlot cattle. J Anim Sci.

[20] NRC (2001). Nutrient Requirements of Dairy Cattle.

[21] Pereira AMF, Baccari FJ, Titto EAL, Almeida JAA (2008). Effect of thermal stress on physiological parameters, feed intake and plasma thyroid hormones concentration in Alentejana, Mertolenga. Frisian and Limousine Int J Biometeorol.

[22] AOAC (1995). Official methods of analysis.

[23] Van Soest PJ, Robertson JB, Lewis BA (1991). Methods of dietary fiber, neutral detergent fiber and non starch polysaccharides in relation to animal nutrition. J Dairy Sci.

[24] Talapatra SK, Ray SC, Sen KC (1940). The analysis of mineral constituents in biological materials. Indian J Vet Sci Anim Husb.

[25] Fenton TW, Fenton M (1979). An improved procedure for the determination of chromic oxide in feed and feces. Can J Anim Sci.

[26] Benzamin MM (1985). Outline of veterinary clinical pathology.

[27] Madesh M, Balasubramanian KA (1998). Microtiter plate assay for superoxide dismutase using MTT reductlion by superoxide. Indian J Biochem Bio.

[28] Brambilla G, Fiori M, Archetti LI (2001). Evaluation of the oxidative stress in growing pigs by microplate assays. J Vet Med Sci.

[29] Lacetera N, Bernabucci U, Ronchi B, Nardone A (1996). Body condition score, metabolic status and milk production of early lactating dairy cows exposed to warm environment. Riv Agr Subtrop Trop.

[30] Purwanto BP, Abo Y, Sakamoto R, Furumoto F, Yamamoto S (1990). Diurnal patterns of heat production and heart rate under thermoneutral conditions in Holstein Friesian cows differing in milk production. J Agricult Sci (Camb).

[31] Singh G, Kamboj ML, Patil NV (2005). Effect of thermal protective measures during hot humid season on productive and reproductive performance of Nili-Ravi buffaloes. Indian Buffalo J.

[32] Chaiyabutr N, Boonsanit D, Chanpongsang S (2011). Effects of cooling and exogenous bovine somatotropin on hematological and biochemical parameters at different stages of lactation of crossbred holstein friesian cow in the tropics. Asian-Australas J Anim Sci.

[33] Upadhyay RC, Singh SV, Kumar A, Gupta SK, Ashutosh A (2010). Impact of climate change on milk production of Murrah buffaloes. Ital J Anim Sci.

[34] Bouraoui R, Lahmar M, Majdoub A, Djemali M, Belyea R (2002). The relationship of temperature-humidity index with milk production of dairy cows in a Mediterranean climate. Anim Res.

[35] Nessim MG (2004). Heat-induced biological changes as heat tolerance indices related to growth performance in buffaloes’. Ph. D. Thesis.

[36] Habeeb AAM, Fatma FIT, Osman SF (2007). Detection of heat adaptability using heat shock proteins and some hormones in Egyptian buffalo calves. Egyptian J Appl Sci.

[37] Chaiyabutr N (1993). Buffalo physiological responses to high environmental temperature and consequences for DAP. Proceedings of Workshop held in conjunction with 6th Asian–Australasian Association of Animal Production Society Congress.

[38] Gudev D, Popova-Ralcheva S, Moneva P, Aleksiev Y, Peeva TZ, Penchev P (2007). Physiological indices in buffaloes exposed to sun. Arch Zootech.

[39] Khongdee T, Sripoon S, Vajrabukka C (2011). The effects of high temperature and wallow on physiological responses of swamp buffaloes (Bubalus bubalis) during winter season in Thailand. J Therm Biol.

[40] Al-Haidary AA (2004). Physiological responses of Naimey Sheep to heat stress challenge under semi-arid environments. Int J Agriculture Biol.

[41] Parmar MS, Madan AK, Rastogi SK, Huozha R (2013). Comparative Study of Seasonal Variations on Hematological Profile in Sahiwal Cows (Bos Indicus) and Murrah Buffalo (Bubalus bubalis). J Anim Res.

[42] Aengwanich W, Kongbuntad W, Boonsorn T (2011). Effects of shade on physiological changes, oxidative stress, and total antioxidant power in Thai Brahman cattle. Int J Biometeorol.

[43] Silva JA, Araújo RD, Júnior AAD, de Brito L, Santos NDFAD, Viana RB (2014). Hormonal changes in female buffaloes under shading in tropical climate of Eastern Amazon. Rev Bras Zootec.

[44] Dhabhar FS (2002). A hassle a day may keep the doctor away: stress and the augmentation of immune function. Integr Comp Biol.

[45] Bishop CR, Athens JW, Boggs DR, Warner HR, Cartwrig GE, Wintrobe MM (1968). Leukokinetic Studies: XIII. A non-steady-state kinetic evaluation of mechanism of cortisone induced granulocytosis. J Clin Invest.

[46] Gangwar PC, Bahga CS, Srivastava RK, Dhingra DP (1982). Effect of spray cooling and wallowing on potassium concentration of erythrocytes in buffaloes (Bos bubalis) during summer. J Agricult Sci.

[47] Kumar A, Singh G, Kumar BV, Meur SK (2011). Modulation of antioxidant status and lipid peroxidation in erythrocyte by dietary supplementation during heat stress in buffaloes. Livest Sci.

[48] Yadav B, Singh G, Wankar A (2015). Adaptive capability as indicated by redox status and endocrine responses in crossbred cattle exposed to different thermal stresses. J Anim Res.

[49] Yadav B, Korde JP (2011). Effect of hyperthermia on antioxidative status of a primary hepatocyte culture. J Adv Vet Res.

[50] Rahal A, Kumar A, Singh V, Yadav B, Tiwari R, Chakraborty S (2014). Oxidative Stress, Prooxidants, and Antioxidants: The Interplay. Biomed Res Int.

[51] Roy KS, Prakash B (2007). Seasonal variation and circadian rhythmicity of the prolactin profile during the summer months in repeat-breeding Murrah buffalo heifers. Reprod Ferti Dev.

[52] Wankar AK, Singh G, Yadav B (2014). Thermoregulatory and adaptive responses of adult buffaloes (Bubalus bubalis) during hyperthermia: Physiological, behavioral, and metabolic approach. Vet World.

